# Characterization and phylogenetic analysis of the complete mitochondrial genome of *Terapon theraps* (Perciformes: Terapontidae)

**DOI:** 10.1080/23802359.2021.1872438

**Published:** 2021-02-11

**Authors:** Lei Zeng, Pimao Chen, Jie Yu, Guobao Chen

**Affiliations:** a South China Sea Fisheries Research Institute, Chinese Academy of Fishery Sciences, Guangzhou, China; b Guangdong Provincial Key Laboratory of Fishery Ecology and Environment, Guangzhou, China; c Scientific Observing and Experimental Station of South China Sea Fishery Resources and Environment Ministry of Agriculture, Guangzhou, China; d Key Laboratory of Marine Ranch Technology, Chinese Academy of Fishery Sciences, Guangzhou, China

**Keywords:** *Terapon theraps*, mitochondrial genome, Terapontidae, phylogenetic analysis

## Abstract

We assembled the complete mitochondrial genome of *Terapon theraps* based on high-throughput Illumina sequencing. The mitochondrial genome of *T. theraps* is 16,587 bp in length, containing 13 protein-coding genes (PCGs), 22 tRNAs, two rRNAs, and one putative control region (CR), and its overall nucleotides base composition is 27.4% A, 25.7% T, 16.9% G, and 29.9% C. Bayesian Inference revealed that all species of Terapontidae formed into one group, while the other nine species within Perciformes clustered into the other group. *Rhynchopelates oxyrhynchus* and *T. theraps* were the closest relatives. These results should help to better understand the phylogenetic interrelationships of Terapontidae.

*Terapon theraps* (Cuvier, 1829) is an omnivorous species that occurs in brackish waters and mangrove habitats of the tropical Indo-west Pacific (Froese and Pauly 2019). It belongs to the family Terapontidae (order Perciformes). In Terapontidae, there are 52 spceises distributed into 16 genera (e.g., *Amniataba* (3)*, Bidyanus* (2)*, Hannia* (1)*, Helotes* (1)*, Hephaestus* (14)*, Lagusia* (1)*, Leiopotherapon* (4)*, Mesopristes* (5)*, Pelates* (3)*, Pelsartia* (1)*, Pingalla* (3)*, Rhynchopelates* (1)*, Scortum* (4)*, Syncomistes* (4)*, Terapon* (3)*, and Variichthys* (2)) (Nelson et al. 2016). However, only a few complete mitochondrial genomes were reported in this family. In this study, we sequenced the mitochondrial genome of *T. theraps*, which helps to better understand the phylogenetics of Terapontidae. This work is essential for taxonomic, systematic, and population genetic studies.

The specimen was collected from estuary of the Pearl River in Guangdong Province, China (E113.793°, N22.503°), in April 2020 and deposited in South China Sea Fisheries Research Institute, Chinese Academy of Fishery Sciences (email: yhr@scsfri.ac.cn) under the voucher number SCSFRI-20200412001. The complete mitochondrial genome was sequenced by next-generation sequencing using the Illumina HiSeq2500 instrument (Illumina, Inc., San Diego, CA, USA) with a *de novo* assembly strategy (Shao et al. 2020). Phylogenetic analysis based on 13 protein-coding genes in mitochondrial genomes was conducted using Bayesian inference in MrBayes v3.2.6 (Ronquist et al. 2012).

The mitochondrial genome of *T. theraps* is 16,587 bp (GenBank Accession number: MW143074) in length, and its overall nucleotides base composition is 27.4% A, 25.7% T, 16.9% G, and 29.9% C. Like other vertebrates (Boore 1999), it has 13 protein-coding genes (PCGs), 22 tRNAs, two rRNAs, and one putative control region (CR). Among all the PCGs, there were 12 (nad2, cox1, cox2, atp8, atp6, cox3, nad3, nad4I, nad4, nad5, cob, and nad1) on the heavy strand, and one (nad6) on the light strand.

We found that the 15 species can be divided into two clades with *Oryzias latipes* (NC_004387) and *Takifugu obscurus* (NC_011626.1) as the outer group (Figure 1). All species of Terapontidae formed into one clade, while the other 9 species clustered into the other clade. *R. oxyrhynchus* and *T. theraps* were found to be the sister species with 94.1% support value. These results confirmed that *T. theraps* belonged to the family Terapontidae and also implied polyphyly nature of the Terapon genus.

**Figure 1.**
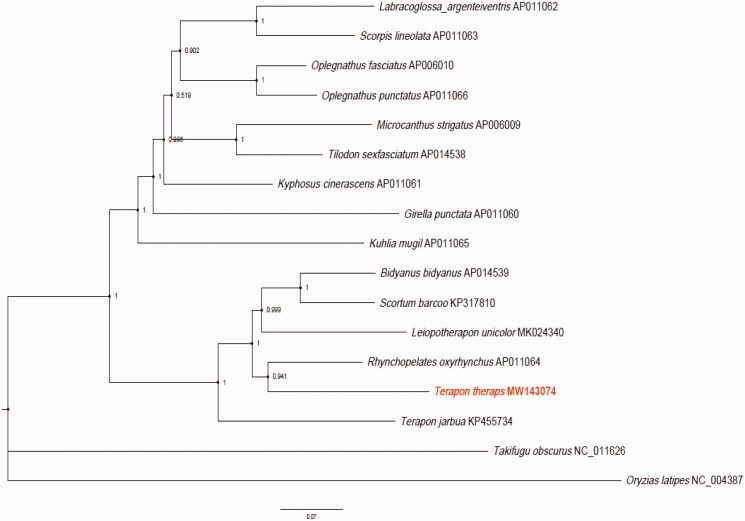
Bayesian Inference phylogeny based on 13 mitochondrial protein-coding genes of *T. theraps* (MW143074) and other 14 species within the Perciformes order. The support values are shown next to the nodes. *Oryzias latipes* (NC_004387) and *Takifugu obscurus* (NC_011626.1) were included as the outgroup taxon.

In conclusion, we sequenced, annotated, and characterized the complete mitogenome of *Terapon theraps*. This research should provide valuable information for exploring genetic diversity and phylogenetic relationships of the Terapontidae family.

## Data Availability

The genome sequence data that support the findings of this study are openly available in GenBank of NCBI at (https://www.ncbi.nlm.nih.gov/) under the accession no MW143074. The associated BioProject, SRA, and Bio-Sample numbers are PRJNA683093, SRR13212380, and SAMN17022208 respectively.
